# Miniscrew-assisted rapid palatal expansion for managing arch perimeter in an adult patient

**DOI:** 10.1590/2177-6709.22.3.097-108.oar

**Published:** 2017

**Authors:** Amanda Carneiro da Cunha, Hisun Lee, Lincoln Issamu Nojima, Matilde da Cunha Gonçalves Nojima, Kee-Joon Lee

**Affiliations:** 1 Universidade Federal do Rio de Janeiro, Department of Pediatric Dentistry and Orthodontics (Rio de Janeiro/RJ, Brazil).; 2 Private Clinic, Seoul, Korea.; 3 Yonsei University,Department of Orthodontics, Seoul, Korea.

**Keywords:** Orthodontic anchorage procedures, Palatal expansion technique, Adult, Malocclusion.

## Abstract

**Introduction:**

Etiology of dental crowding may be related to arch constriction in diverse dimensions, and an appropriate manipulation of arch perimeter by intervening in basal bone discrepancies cases, may be a key for crowding relief, especially when incisors movement is limited due to underlying pathology, periodontal issues or restrictions related to soft tissue profile.

**Objectives:**

This case report illustrates a 24-year old woman, with maxillary transverse deficiency, upper and lower arches crowding, Class II, division 1, subdivision right relationship, previous upper incisors traumatic episode and straight profile. A non-surgical and non-extraction treatment approach was feasible due to the miniscrew-assisted rapid palatal expansion technique (MARPE).

**Methods:**

The MARPE appliance consisted of a conventional Hyrax expander supported by four orthodontic miniscrews. A slow expansion protocol was adopted, with an overall of 40 days of activation and a 3-month retention period. Intrusive traction miniscrew-anchored mechanics were used for correcting the Class II subdivision relationship, managing lower arch perimeter and midline deviation before including the upper central incisors.

**Results:**

Post-treatment records show an intermolar width increase of 5 mm, bilateral Class I molar and canine relationships, upper and lower crowding resolution, coincident dental midlines and proper intercuspation.

**Conclusions:**

The MARPE is an effective treatment approach for managing arch-perimeter deficiencies related to maxillary transverse discrepancies in adult patients.

## RESUMO

**Introdução:**

a etiologia do apinhamento dentário pode estar relacionada à constrição das arcadas dentárias em diversas dimensões, e a manipulação apropriada do perímetro da arcada, por meio da intervenção em casos de discrepâncias de base óssea, pode ser o fator chave para a dissolução do apinhamento, especialmente em casos onde a movimentação de incisivos é limitada em decorrência de problemas periodontais ou restrições relacionadas ao perfil facial.

**Objetivos:**

o presente relato de caso ilustra uma paciente de 24 anos de idade, com deficiência transversa de maxila, apinhamento das arcadas superior e inferior, má oclusão de Classe II, 1^a^ divisão, subdivisão direita, incisivos superiores previamente traumatizados e perfil reto. A abordagem de tratamento não-cirúrgica e sem extrações foi viável devido à técnica de expansão rápida da maxila assistida por mini-implantes (MARPE, do inglês *miniscrew-assisted rapid palatal expansion*).

**Métodos:**

o dispositivo MARPE foi confeccionado a partir de um expansor Hyrax convencional apoiado em quatro mini-implantes. Foi adotado o protocolo de expansão lenta, com um período total de ativações de 40 dias e 3 meses de contenção. Uma mecânica de tração intrusiva apoiada em mini-implantes foi utilizada para a correção da relação de Classe II subdivisão direita, adequação do perímetro da arcada inferior e correção do desvio da linha média antes da inclusão dos incisivos centrais superiores.

**Resultados:**

os registros pós-tratamento demonstraram o aumento de 5,0 mm na distância intermolares, relação bilateral de Classe I de molares e caninos, resolução do apinhamento superior e inferior, linhas médias dentárias coincidentes e intercuspidação adequada.

**Conclusões:**

a técnica MARPE é uma abordagem de tratamento efetiva para a resolução da deficiência de perímetro das arcadas dentárias relacionada à discrepância maxilar transversa em pacientes adultos.

## INTRODUCTION

Maxillary arch constriction derived from an underlying transverse deficiency is a common etiologic factor associated to dental crowding or protrusion.^1^ Therefore, improvement of the sagittal arch dimension may play an important role for solving arch perimeter problems,^1^ especially when additional factors such as previous traumatic injuries, pathologies and soft tissue profile restrictions limit the decision for extraction approaches.

The maxillary transverse deficiency has been successfully treated in young patients by intervention on the midpalatal suture for separating the maxillary bones with the rapid palatal expansion technique (RPE).^2^ In addition to the midpalatal suture, exerted forces must counteract the resistance provided by circumaxillary sutures and structures^2^ ^-^ ^4^ such as zygomaxillary buttress and sphenoidal structures.^4^ ^,5^ Therefore, potential alveolar bending and dental tipping^6^ is expected from orthopedic forces exerted in adult patients, due to the progressively interdigitated suture pattern and increased stiffness of surrounding structures as skeletal maturity advances. Consequently, tooth resorption, periodontal damages,^7^ ^-^ ^9^ failure or limited expansion,^10^ questionable long-term stability,^11^ soft tissue swelling and ulcerations^12^ commonly results from conventional palatal expansion technique carried out in mature patients. 

In order to overcome dentoalveolar undesirable effects and maximize skeletal expansion potential, a nonsurgical miniscrew-assisted rapid palatal expansion technique (MARPE), was introduced^13^ and recently demonstrated successful outcomes by providing effective midpalatal suture splinting in adult patients.^14^ The aim of this case report was to present a non-extraction adult treatment conducted with the MARPE technique.

## DIAGNOSIS

A 24-year old woman attended the Orthodontic Department at Yonsei University with the chief complaint related to upper right canine position and dental crowding. No medical complications and history of trauma was reported. Facial analysis showed a symmetrical face, balanced facial thirds and an pleasant soft tissue profile. 

**Figure 1:**
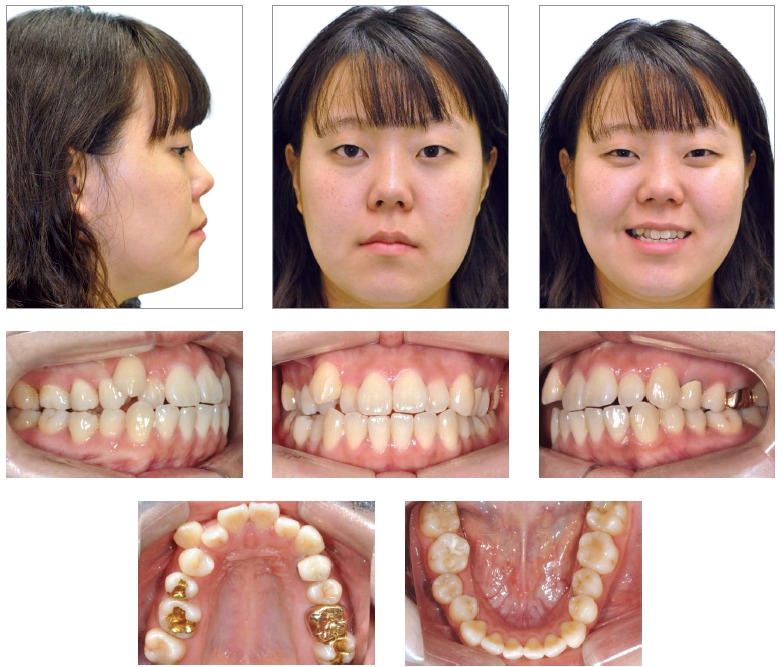
Pretreatment facial and intraoral photographs.

**Figure 2:**
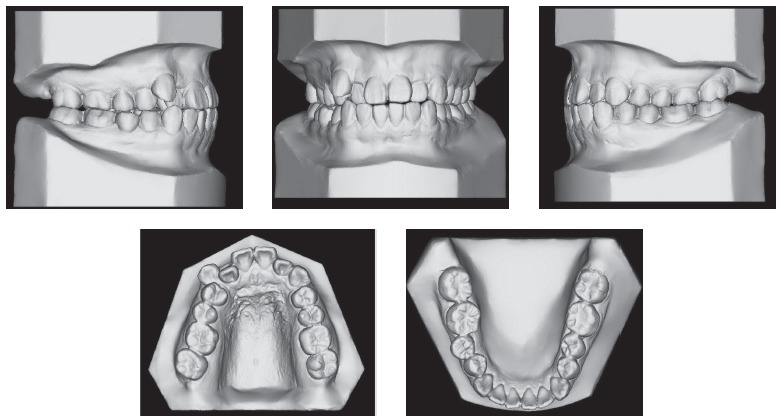
Pretreatment digital dental casts.

Upper dental midline was coincident and lower dental midline was 1.0 mm deviated to the right side in relation to the sagittal facial plan. Intraoral clinical examination and dental casts analysis revealed a transverse maxillary deficiency expressed by an edge to edge occlusion of upper right first premolar and molar, and upper lateral incisors and right second premolar crossbites; a Class II, division 1, subdivision right relationship; proclined lower incisors; edge to edge overjet, 0-mm overbite and noticeable mobility of upper central incisors. Maxillary and mandibular arch length discrepancies were 8.5 mm and 0.5 mm, respectively; and the difference between upper and lower first intermolar widths was 5.2 mm (Figs 1 and 2).

**Figure 3:**
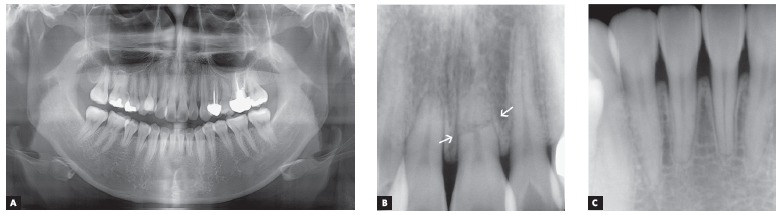
A) Pretreatment panoramic radiograph. B, C) Upper and lower incisors periapical radiographs. White arrows indicate the apical root fracture of the upper left central incisor.

**Figure 4:**
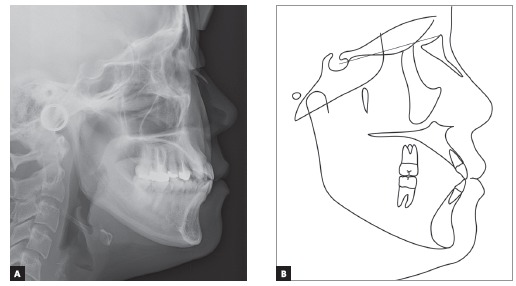
A) Pretreatment cephalometric radiograph and B, pretreatment cephalometric tracing.

 Radiographic analysis indicated an endodontic treatment in the maxillary left first premolar and molar, reduced root length of upper central incisors and lower right second premolar; and a complete fracture line on maxillary left central incisor located in the apical third of the root, due to unknown cause (Fig 3). Cephalometric analysis showed a Class I (ANB = 2.3^o^) normodivergent skeletal pattern (SN:GoMe = 35.9^o^), well-positioned upper incisors (U1:SN = 106.2^o^), and proclined lower incisors (L1:NB = 32.7^o^) (Fig 4) (Table 1). 

**Table 1:** Pretreatment and post-treatment cephalometric measurements.

	Pretreatment	Post-treatment
SNA (degrees)	82,6	81,3
SNB (degrees)	80,3	78,9
ANB (degrees)	2,3	2,4
SN:GoMe (degrees)	35,9	36,5
U1:SN (degrees)	106,2	106,8
L1:NB (degrees)	32,7	32,4
IMPA (degrees)	96,5	96,8
Ocl:SN (degrees)	16,8	19,2
Upper lip to S line (mm)	-0,2	0,7
Lower lip to S line (mm)	+1,1	+1,5

## TREATMENT OBJECTIVES AND ALTERNATIVES

The treatment objectives were to:

1) Correct the transverse discrepancy.
2) Manage upper and lower arch discrepancies.
3) Consider a solution for managing traumatized upper incisors.
4) Establish a bilateral Class I molar and canine relationship, proper overjet, overbite and correct dental midline.

Mild and severe arch length discrepancies, as observed in lower and upper arches, respectively, could be solved by extracting four premolars or the upper central incisors and lower second premolars. The considerable amount of retraction of previously traumatized incisors, as well as the negative impact on patient’s smile and soft tissue profile esthetics related to the former and later alternatives, supported the decision for a non-extraction treatment approach. Alternatively, an asymmetric extraction of upper right first premolar was not adopted due to the concerns on the arch constriction and development of a posterior crossbite on the right side.

A surgically-assisted rapid palatal expansion technique (SARPE) reveals to be an alternative for correcting skeletal transverse discrepancies in adult patients. However, due to increased overall treatment cost and potential complications of a surgical procedure, this option was also disregarded from the treatment plan. 

In order to substantiate the option for a non-surgical approach, an efficient alternative should provide skeletal expansion with minimum dentoalveolar side effects. Therefore, a miniscrew-assisted rapid palatal expansion (MARPE) technique was considered for this case, as besides correcting transverse discrepancy, the skeletal maxillary expansion would also provide an increase in upper arch length, for crowding solution. 

## TREATMENT PROGRESS

**Figure 5:**
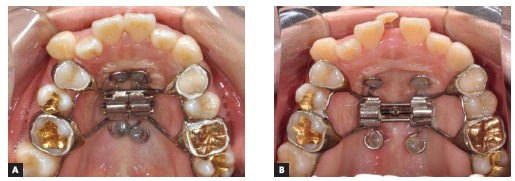
MARPE appliance: A) immediately after placement and B) at the end of the activation period.

**Figure 6:**
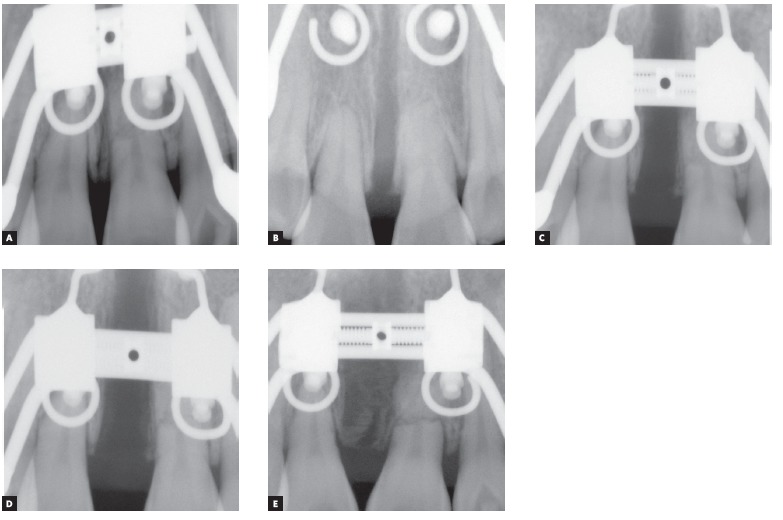
Upper incisors periapical radiographs: A) before expansion, B-D) during active expansion and E) after activation period.

**Figure 7:**
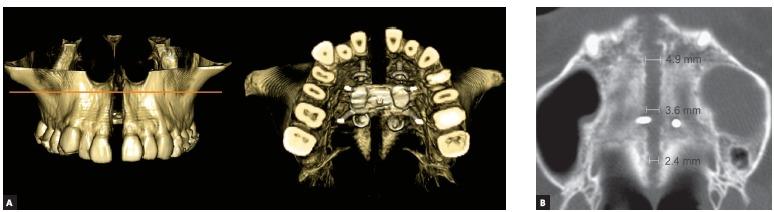
CBCT immediately after MARPE: A) frontal and occlusal views of tridimensional volumetric rendering, and B) axial slice with linear measurements of anterior, intermediate and posterior midpalatal widths.

The MARPE appliance, previously described by Lee et al,^13^ 2010, was produced by transferring the first molars and premolars bands to the patient’s impression for further adapting a conventional Hyrax expander in the plaster cast. Then, four stainless steel wire hooks were passively adapted on the palate and soldered on the base of the Hyrax screw, located anteriorly in the palatal rugae and posteriorly in the parasagittal area. After adapting and cementing the MARPE appliance, four orthodontic miniscrews (1.8 mm diameter x 8-mm and 7-mm length, for anterior and posterior regions, respectively) (Orlus, Ortholution, Seoul, Korea) were placed in the center of the hooks under local anesthesia and covered by a light-cured composite (Transbond XT, 3M Unitek, Monrovia, Calif, USA). The activation protocol was one-quarter of a turn (0.2 mm) once a day, with an overall activation period of 40 days and a 3-month retention period (Fig 5). Midpalatal suture splitting was confirmed with intraoral radiographs (Fig 6) and a cone-beam computed tomography (CBCT) (Fig 7). 

At the end of retention period, 0.018 x 0.025-in preadjusted brackets (Formula-R, Tomy Inc, Tokyo, Japan) were bonded to mandibular and maxillary arches, with the exception of the maxillary central incisors, right lateral incisor and canine. As advised by the oral surgeon, in order to prevent dental infections or any inflammatory process, the apical third of the upper left central incisor root was surgically removed. 

**Figure 8:**
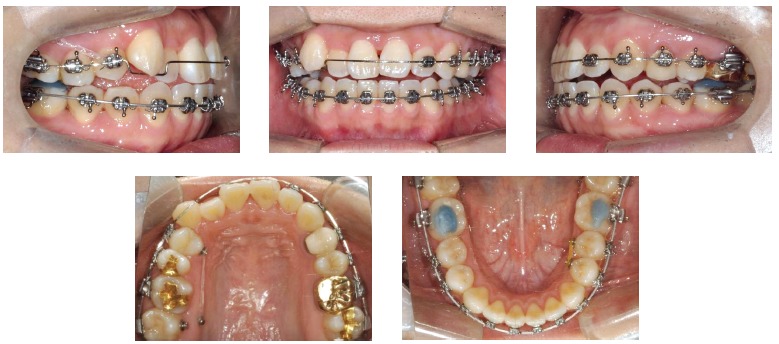
Intraoral photographs of treatment progress.

Alignment and leveling phase proceeded with a sequence of 0.012-in, 0.014-in NiTi, 0.016-in NiTi and 0.016 x 0.022-in NiTi sectional and continuous wires, for maxillary and mandibular arches, respectively. Then, four miniscrews (diameter, 1.8 mm; length, 7 mm) (Orlus, Ortholution, Seoul, Korea) were placed, two into the buccal and palatal alveolar bone between upper right first molar and second molar; and the other two into the buccal alveolar bone between lower second premolars and first molars, one at each right and left side. An intrusive traction mechanics with elastomeric chains (150 gF) was applied for correcting the Class II subdivision relationship, managing lower arch perimeter and correcting midline deviation (Fig 8) to the left side by managing the spaces raised from the miniscrew force system. Subsequently, a light-cured composite resin (Light-Core, Bisco Inc., Illinois, USA) was placed on lower first molars for bite raising. Maxillary central incisors, right lateral incisor and canine were bonded and progressively included in the archwire. Finalization phase proceeded with 0.016 x 0.022-in SS archwires retaining anterior root torque of the upper incisor teeth. The appliance was removed after 32 months of treatment, fixed lingual retainers were bonded to upper and lower anterior teeth and a removable circumferential retainer was placed on the maxillary arch for 24 hours/day use during the first three months and at night period for the following nine months.

## TREATMENT RESULTS

**Figure 9:**
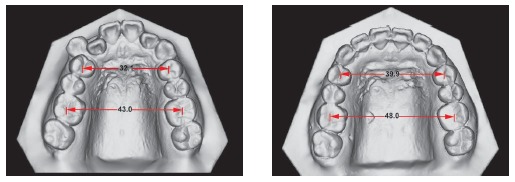
Pretreatment and post-treatment inter-premolar and intermolar widths measured on maxillary digital dental casts.

**Figure 10:**
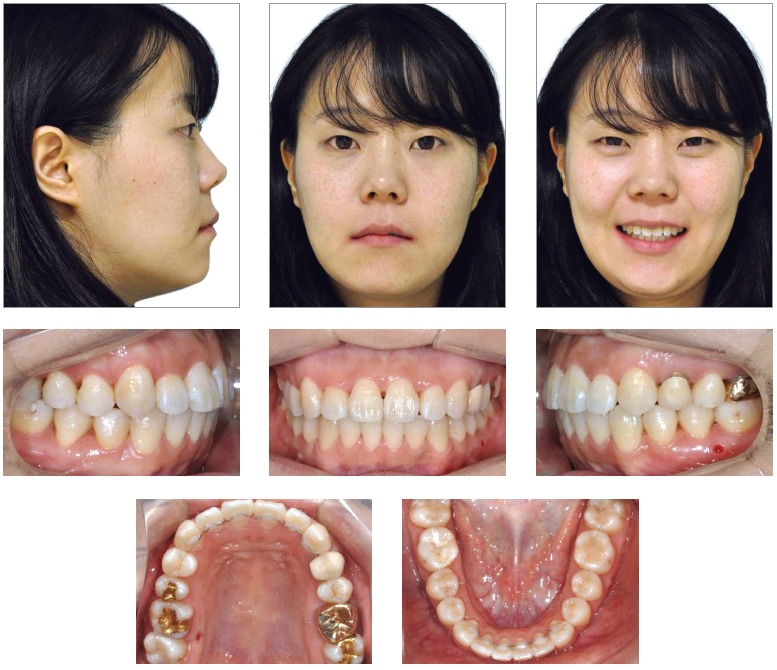
Post-treatment facial and intraoral photographs.

**Figure 11:**
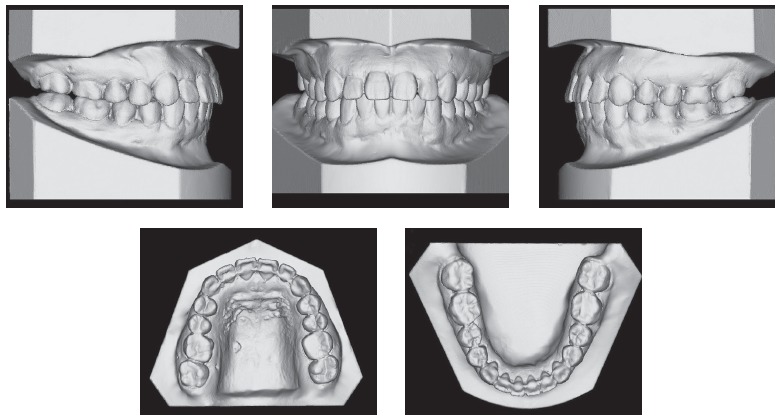
Post-treatment digital dental casts.

**Figure 12:**
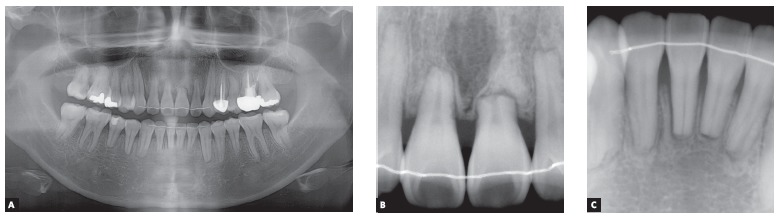
A) Post-treatment panoramic radiograph. B, C) Upper and lower incisors periapical radiographs.

**Figure 13:**
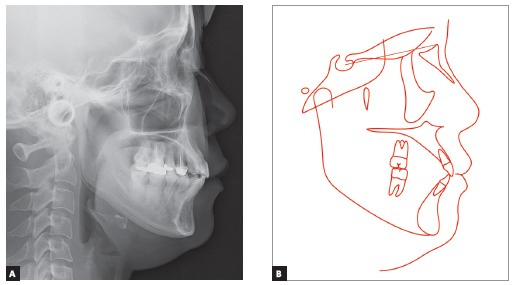
A) Post-treatment cephalometric radiograph and B) cephalometric tracing.

**Figure 14:**
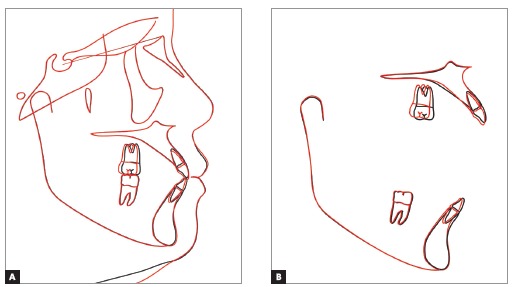
Superimposition of cephalometric tracings of pretreatment (black line) and post-treatment (red line): A) superimposed on sella-nasion plane at sella and B) superimposed on palatal and mandibular planes.

**Figure 15:**
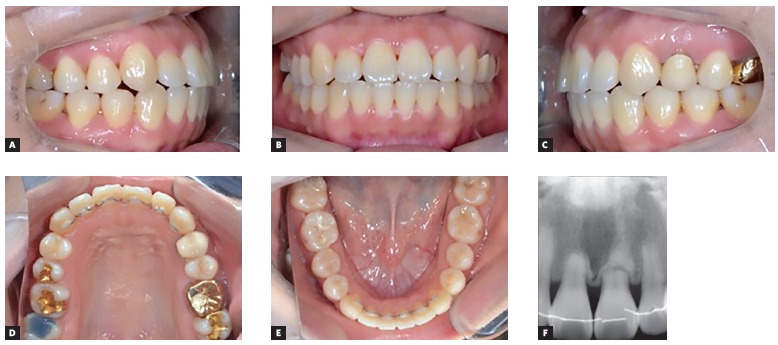
A-E) 3-year follow-up intraoral photographs and F) 2-year upper incisors periapical radiograph.

The maxillary transverse deficiency was solved with an increase of 7.8 mm and 5 mm in the maxillary first premolars and first molars width, respectively (Fig 9). Final treatment photographs and dental casts revealed a bilateral Class I molar and canine relationships, upper and lower crowding resolution, coincident dental midlines and proper intercuspation (Figs 10 and 11). Panoramic and periapical radiographs showed a mild upper and lower incisor’s apical root resorption, although, periodontal tissues soundness was preserved. On the other hand, the lower right second premolar root length was unaltered (Fig 12). Cephalometric outcomes and tracing superimpositions indicated the maintenance of the mandibular plane angle (SN:GoMe = 36.5^o^), upper (U1:SN = 106.8^o^) and lower incisors (L1:NB = 32.4^o^) inclinations, and intrusive retraction of upper right first molar (Figs 13 and 14). Soft tissue facial profile was maintained and smile esthetics was improved. The results remained stable over the 3-year follow-up clinical photographs and 2-year upper incisors periapical radiograph (Fig 15). 

## DISCUSSION

The SARPE provides the correction of transverse discrepancies in adult patients by surgical osteotomies of the zygomaticomaxillary buttress, midpalatal suture and in some techniques, also by releasing the pterygoid plates.^15^ ^,16^ However, it has been shown that the midpalatal suture hardly fuses in subjects in their young adulthood.^17^ Related to this notion, transverse discrepancies smaller than 5 mm are considered eligible for orthodontic camouflage, through orthopedic forces, in skeletal mature patients.^6^ However, potential periodontal damage that may arise from this treatment approach constitutes an important clinical concern. 

Evidences from computed tomography (CT) and cone-beam computed tomography (CBCT) studies showed that tipping and bodily movement of anchor teeth, reduction of alveolar bone height and thickness, bone dehiscence and gingival recession may result from tooth-borne or Hyrax type; and tooth-tissue-borne or Haas type expanders, even when performed in growing patients.^18^ ^,19^ Therefore, the MARPE technique was considered as a suitable option for this adult case since it is based on a tooth-bone-borne appliance that transmits expansion forces to basal bones by a miniscrew anchorage system, providing greater skeletal expansion and also adequate structural stiffness for maintaining the amount of expansion during the consolidation phase.^13^

The present case illustrates a successful maxillary expansion in a young adult with a complete disjunction of the midpalatal suture from the anterior nasal spine to posterior nasal spine, classified as type I palatal split pattern.^20^ The palatal split pattern has been also evaluated for SARPE technique, and some previous literature on SARPE shows the prevalence of a type II pattern, consisting of an incomplete disjunction of the midpalatal suture.^20^ Furthermore, the achievement of a type I pattern has been associated to an additional surgical releasing of pterygoid plates.^15^ ^,16^ The type I palatal split pattern achieved with the MARPE suggests that, despite the absence of any surgical osteotomy, the position of posterior miniscrews may have an important role on providing adequate stress distribution, favoring the complete disjunction of the midpalatal suture. However, further studies still need to be conducted on this issue.

A recently published study regarding the clinical efficacy and stability of the MARPE technique conducted on 69 subjects ranging from 19 to 26 years old, reported a success rate of 86.96%, maintenance of skeletal and dentoalveolar expansion and periodontal structures soundness during retention period.^14^ Similarly, the amount of expansion obtained in the present report has been retained until the last follow-up records of 3-years after debonding. Regardless a considerable inter-individual variability was found in the midpalatal inter-digitation and obliteration parameters,^17^ ^,21^ a slow expansion protocol, with activation of one-quarter of a turn per day was considered with the main purpose of allowing adequate tissues adaptation to exerted forces and minimizing patient’s discomfort, especially due to increased maxillary bone stiffness with age.^17^ ^,22-25^

A non-extraction treatment approach was possible due to the arch length increase provided by both maxillary expansion and monocortical miniscrew mechanics. A combined intrusive and retraction system was applied to the maxillary right segment in order to correct the Class II subdivision relationship^26^ as well as provide enough space for upper teeth alignment. The study performed by Bechtold et al,^26^ 2013, which investigated and discussed the distalization pattern of maxillary arch according to the linear force vectors provided by interradicular monocortical miniscrews, showed that the expected clinical outcome is directly related to the line of force action according to the center of resistance of the maxillary arch. In this case, the miniscrew was placed into the buccal and palatal alveolar bone between the upper right first molar and second molar, thus favoring the horizontal resultant force vector, and consequently, the distal translation of the maxillary right arch segment. As demonstrated by the tracing superimpositions, upper incisors presented minimal displacement at incisal edge level and, unlike previous reports,^27^ ^,28^ mandibular plane angle was not increased, possibly due to elimination of cuspal interference during the transverse expansion and vertical displacement control provided by the intrusive retraction mechanics.

Root resorption, loss of vitality and pulp calcifications may arise from orthodontic treatment of previously traumatic injured teeth.^29^ In the present case, the orthodontic movement of previously root fractured maxillary left central incisor entailed the risk of a further root shortening due to the orthodontic induced root resorption of the coronal fragment.^30^ Therefore, the maxillary left central incisor was monitored during the initial phase of the treatment, and due to the absence of any radiographic and clinical signs of complications such as pulp necrosis or granulation tissue,^30^ it was bonded and included in the upper archwire under light forces and for the shortest period of time as possible. Despite the non-extraction treatment approach with minor movement of upper incisor teeth, mild tooth resorption of incisor’s apex was noticed at the end of active orthodontic treatment. Therefore, patient has been constantly radiographically and clinically monitored regarding the anterior teeth’s vitality, root resorption and periodontal status.

## CONCLUSIONS

The MARPE is a clinical effective technique for correction of transverse discrepancies in skeletal mature patients as it provides maxillary expansion at sutural levels and decrease dentoalveolar side effects. It should be considered as an alternative for managing arch perimeter length, especially in limited adult orthodontic treatments.
